# Development of a luciferase-based biosensor to assess enterovirus 71 3C protease activity in living cells

**DOI:** 10.1038/s41598-017-10840-x

**Published:** 2017-09-04

**Authors:** Yuan Zhang, Xianliang Ke, Caishang Zheng, Yan Liu, Li Xie, Zhenhua Zheng, Hanzhong Wang

**Affiliations:** 1 Key Laboratory of Special Pathogens and Biosafety, Center for Emerging Infectious Diseases, Wuhan Institute of Virology, Chinese Academy of Sciences, Wuhan, 430071 China; 2 Guangzhou Institute of Pediatrics, Guangzhou Women and Children Medical Center, Guangzhou, 510623 China

## Abstract

Enterovirus 71 (EV71) is a major pathogen of hand, foot, and mouth disease (HFMD). To date, no antiviral drug has been approved to treat EV71 infection. Due to the essential role that EV71 3 C protease (3C^pro^) plays in the viral life cycle, it is generally considered as a highly appealing target for antiviral drug development. In this study, we present a transgene-encoded biosensor that can accurately, sensitively and quantitatively report the proteolytic activity of EV71 3C^pro^. This biosensor is based on the catalyzed activity of a pro–interleukin (IL)-1β-enterovirus 3C^pro^ cleavage site-*Gaussia* Luciferase (GLuc) fusion protein that we named i-3CS-GLuc. GLuc enzyme is inactive in the fusion protein because of aggregation caused by pro–IL-1β. However, the 3C^pro^ of EV71 and other enteroviruses, such as coxsackievirus A9 (CVA9), coxsackievirus B3 (CVB3), and poliovirus can recognize and process the canonical enterovirus 3C^pro^ cleavage site between pro–IL-1β and GLuc, thereby releasing and activating GLuc and resulting in increased luciferase activity. The high sensitivity, ease of use, and applicability as a transgene in cell-based assays of i-3CS-GLuc biosensor make it a powerful tool for studying viral protease proteolytic events in living cells and for achieving high-throughput screening of antiviral agents.

## Introduction

Human enterovirus 71 (EV71) is the major pathogen of herpangina and hand, foot and mouth disease, particularly affecting children and infants^1^. In severe infection cases, EV71 can damage the central nervous system (CNS), leading to viral meningitis, encephalitis, myocarditis and pulmonary edema with high fatality^2^. EV71 was first identified in California in 1969^3^. In the past three decades, EV71 epidemics have been observed in China, Australia, the United States, Germany, Malaysia, etc.^4–8^, causing serious threats to global public health. In 1998, a severe outbreak of EV71 in Taiwan caused approximately 129,000 cases including 405 severe cases and 78 deaths^8^. From March 2008 - June 2009, more than 600,000 HFMD cases and 126 deaths were reported in China^9^. Although an inactivated vaccine was developed recently, its efficacy and safety require further testing^10^. Unfortunately, no approved direct-acting antiviral drug is available for EV71 infection to date. Development of antiviral agents represents an urgent unmet need for EV71 control.

EV71 belongs to the genus *Enterovirus* of the *Picornaviridae* family, with a positive sense, single-stranded RNA genome approximately 7400 nt in length. The genomic RNA encodes a large and single precursor polyprotein^11^. The precursor is then processed into three polyproteins: P1, P2, and P3^12^. P1 is further cleaved into four structural proteins (VP1, VP2, VP3, and VP4). P2 and P3 are proteolytically cleaved into seven nonstructural proteins (2 A, 2B, 2 C, 3 A, 3B, 3 C, and 3D) during viral infection and replication^13^. Among the viral proteins, 3 C protease (3C^pro^) is essential for precursor and polyproteins processing, RNA binding, and viral replication^14, 15^. Thus, 3C^pro^ is generally considered to be an appealing target for anti-EV71 drug development. Sensitive and effective screening to identify the chemical compounds or unpurified natural products that inhibit 3C^pro^ activity is a key technology for EV71 treatment.

Luciferase (Luc) refers to a class of oxidative enzymes that catalyze specific luciferin substrates to produce bioluminescence. Several luciferases require no post-translational processing for enzymatic activity and show a linear relationship between concentration and their resulting bioluminescence^16, 17^. These properties render them excellent genetic reporters. Luc-fused proteins can be easily quantified by measuring their catalyzed bioluminescence with a luminometer, providing the detection sensitivity up to femtogram level^18^. *Gaussia* Luciferase (GLuc) is a naturally secreted luciferase from the deep sea copepod *Gaussia Princeps* ^19^. Deletion of the N-terminal secretion signal peptide of GLuc made it intracellularly retained but did not affect its catalytic activity^20^. In 2013, scientists found that the fusion of mouse pro–interleukin (IL)-1β on the N-terminal of GLuc lacking its secretion signal peptide can inhibit its catalytic activity because pro–IL-1β possesses a strong propensity to form protein aggregates, and based on this, they developed a biosensor pro–IL-1β-GLuc (iGLuc) that can report the proteolytic activity of caspase-1 in the course of inflammasome activation in a highly sensitive manner^21^.

**Figure 1:**
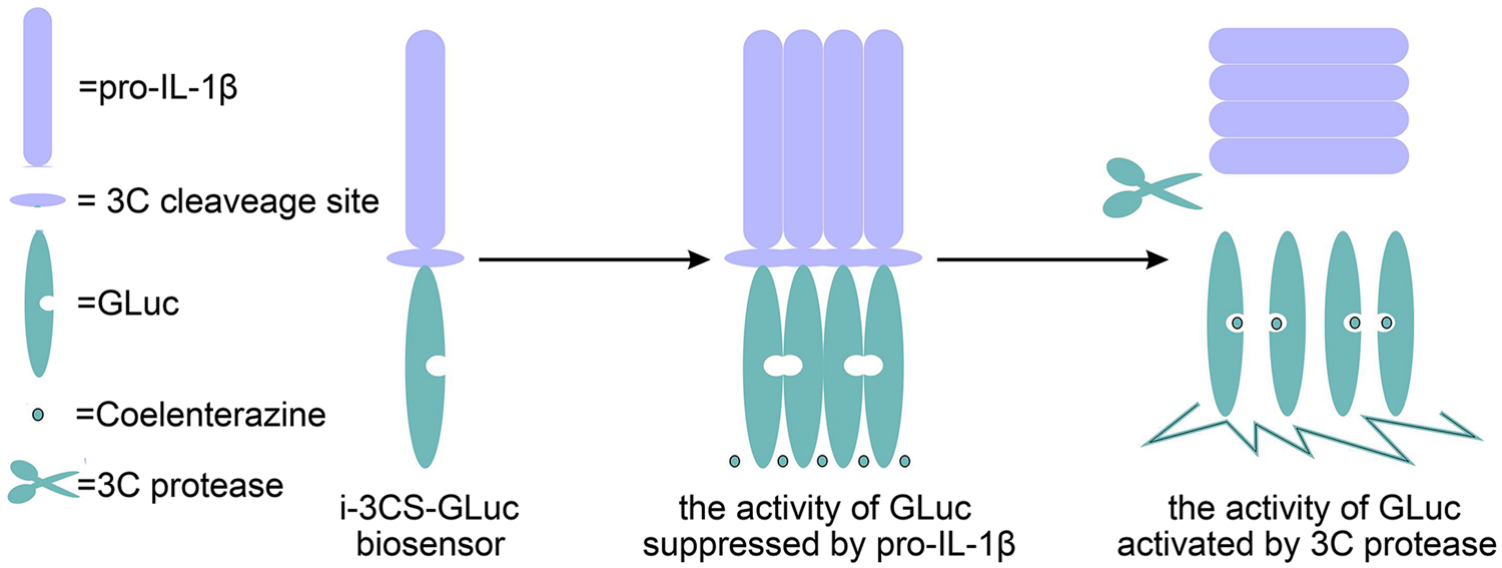
Working principle of the luciferase-based biosensor for EV71 3C^pro^ activity. The biosensor i-3CS-GLuc consists of mouse pro–interleukin (IL)-1β, an EV71 3C protease cleavage site and *Gaussia* luciferase (GLuc) lacking N-terminal secretion signal peptide. In living cells, GLuc activity is suppressed by protein aggregation caused by pro–IL-1β. EV71 3C^pro^ can recognize and process the cleavage site between pro–IL-1β and GLuc, thus releasing and activating the GLuc enzyme.

In this study, we report a quantifiable and sensitive transgene-encoded biosensor that monitors the activity of EV71 3C^pro^ in living cells. As shown in Fig. 1, a canonical enterovirus 3C^pro^ cleavage site EALFQ↓GPPK was inserted between mouse pro–IL-1β and GLuc lacking its secretion signal peptide to generate the biosensor pro–IL-1β-enterovirus 3C^pro^ cleavage site-GLuc (i-3CS-GLuc). Within this biosensor, the GLuc enzyme is normally inactive because of the protein aggregation caused by pro–IL-1β. We demonstrated that the 3C^pro^ of EV71 and some other enteroviruses could recognize and process the EALFQ↓GPPK site within i-3CS-GLuc, thus activating the GLuc enzyme and allowing the monitoring of cytosolic cleavage events and protease activity with high sensitivity and specificity. Moreover, this biosensor’s ease of use and its applicability in living cells make it a powerful tool to screen antiviral drugs with high-throughput.

## Results

### Construction and characterization of the Luc-based biosensor i-3CS-GLuc for EV71 3C^pro^ activity

**Figure 2:**
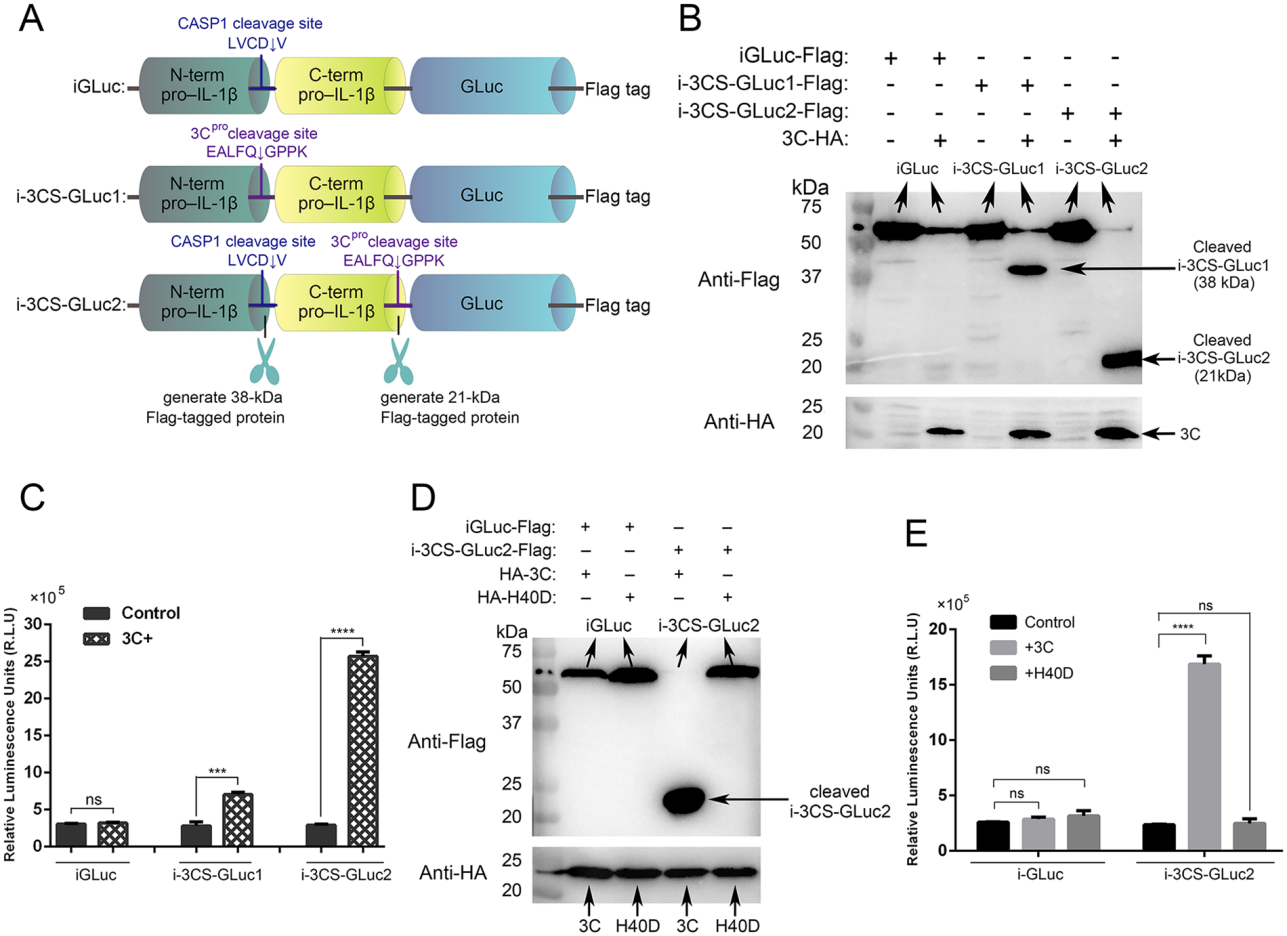
Construction and characterization of the luciferase-based biosensor for EV71 3C^pro^ activity. (**A**) Construction of iGLuc^21^, i-3CS-GLuc1, and i-3CS-GLuc2. (**B**) Cleavage of iGLuc, i-3CS-GLuc1 and i-3CS-GLuc2 by EV71 3C^pro^ detected by Western blotting assays. (**C**) Cleavage of iGLuc, i-3CS-GLuc1, and i-3CS-GLuc2 by EV71 3C^pro^ detected by luciferase assays. The results are presented as the means ± standard deviations (SD) of triplicate measurements (Student’s t test; ns, nonsignificant, ***p < 0.001, ****p < 0.0001). (**D**) Cleavage of iGLuc and i-3CS-GLuc2 by wild-type EV71 3C^pro^ and the catalytically inactive 3C^pro^ mutant H40D detected by Western blotting assays. The blots of Anti-HA were cropped and the full-length blots were displayed in Supplementary Information. (**E**) Cleavage of iGLuc and i-3CS-GLuc2 by wild typed EV71 3C^pro^ and the catalytically inactive 3C^pro^ mutant H40D detected by luciferase assay. The results are presented as the means ± SD of triplicate measurements (Student’s t test; ns, nonsignificant, ****p < 0.0001).

As shown in Fig. 2A, a fusion protein construct of mouse pro–IL-1β and GLuc lacking its N-terminal secretion signal (iGLuc) was generated as described in a previous report^21^ to serve as the negative control in this study. To generate biosensors for EV71 3C^pro^ activity, the canonical enterovirus 3C^pro^ cleavage site EALFQ↓GPPK was inserted into the iGLuc construct at different positions. We replaced the caspase-1 cleavage site LVCD↓V within mouse pro–IL-1β with enterovirus 3C^pro^ cleavage site EALFQ↓GPPK to generate the i-3CS-GLuc1 construct and inserted the EALFQ↓GPPK site between mouse pro–IL-1β and GLuc to generate the i-3CS-GLuc2 construct.

As shown in Figs 2B and S1, the co-expressed EV71 3C^pro^ could recognize the EALFQ↓GPPK site in both biosensors. It cleaved i-3CS-GLuc1 to generate an approximately 38-kDa Flag tagged protein and cleaved i-3CS-GLuc2 to generate an approximately 21-kDa Flag tagged protein due to the different positions the EALFQ↓GPPK site inserted. In contrast, the negative control iGLuc, which included the caspase-1 cleavage site but did not include the 3C^pro^ cleavage site, was cleaved by mouse caspase-1 but was not cleaved by 3C^pro^. These results indicated that EV71 3C^pro^ could recognized and processed the EALFQ↓GPPK site within both i-3CS-GLuc biosensors effectively and specifically.

Luciferase assays were performed to validate the efficiency of our biosensors. As shown in Fig. 2C, with or without EV71 3C^pro^, the negative control iGLuc generated similar bioluminescence of (3.06 ± 0.05) × 10^5^ and (3.20 ± 0.08) × 10^5^ (N = 3) in HEK293T cells. In contrast, when we co-expressed EV71 3C^pro^ with our biosensors, which led to the cleavage of fusion proteins, bioluminescence increased significantly. For i-3CS-GLuc1, the signal increased from (2.82 ± 0.31) × 10^5^ to (7.04 ± 0.18) × 10^5^ (N = 3) in the presence of EV71 3C^pro^. For i-3CS-GLuc2, a more significant increase of GLuc signal from (2.92 ± 0.06) × 10^5^ to (25.71 ± 0.34) × 10^5^ (N = 3) was measured. These results indicate that EV71 3C^pro^-mediated cleavage relieved the suppression of GLuc catalytic activity by pro–IL-1β in both biosensors. Thus, both biosensors can monitor the activity of EV71 3C^pro^. However, the i-3CS-GLuc2 construct showed a higher sensitivity and signal to background ratio (SBR). Because no identified conformation analysis result was available for full length mouse pro–IL-1β or GLuc, the conformation of i-3CS-GLuc constructs were predicted by I-TASSER server mainly based on homology to mature human IL-1β using default parameter^22–24^. As shown in the top predicted models (Fig. S2), the 3C^pro^ cleavage site in i-3CS-GLuc2 is exposed on the surface, whereas the 3C^pro^ cleavage site in i-3CS-Gluc1 is partially surrounded by other domains of i-3CS-Gluc1, which may prevent the access of 3C^pro^. Since the threading templates used for prediction has only 33% coverage of the full length of i-3CS-GLuc proteins, the quality of predicted models is low (C-score = −3.32 and −3.72, typically C-score is in the range of [−5, 2], a higher C-core implicates higher confidence of prediction). However, these predicted models indicated that the 3C^pro^ cleavage site is more accessible in i-3CS-GLuc2 than in i-3CS-GLuc1, which may lead to greater cleavage and explain the increased sensitivity of i-3CS-GLuc2. Thus, i-3CS-GLuc2 was used for the subsequent experiments.

To confirm the specificity of our biosensor, a catalytically dead EV71 3C^pro^ mutant H40D was used. H40 is an essential component of the catalytic triad of EV71 3C^pro^ ^25^. H40D substitution in the active site of EV71 3C^pro^ disrupts its protease activity. Just as expected, the result of WBs showed that EV71 3C^pro^ H40D could not cleave i-3CS-GLuc2 (Fig. 2D). As shown in Fig. 2E, the co-expression of EV71 3C^pro^ with i-3CS-GLuc2 obviously increased bioluminescence, whereas the co-expression of EV71 3C^pro^ H40D with i-3CS-GLuc2 minimally affected bioluminescence, indicating that catalytically inactive 3C^pro^ is incapable of activating our biosensor and confirming that i-3CS-GLuc2 can monitor the proteolytic cleavage event precisely.

### Monitoring the inhibition of EV71 3C^pro^ activity by Rupintrivir using the i-3CS-GLuc2 biosensor

**Figure 3:**
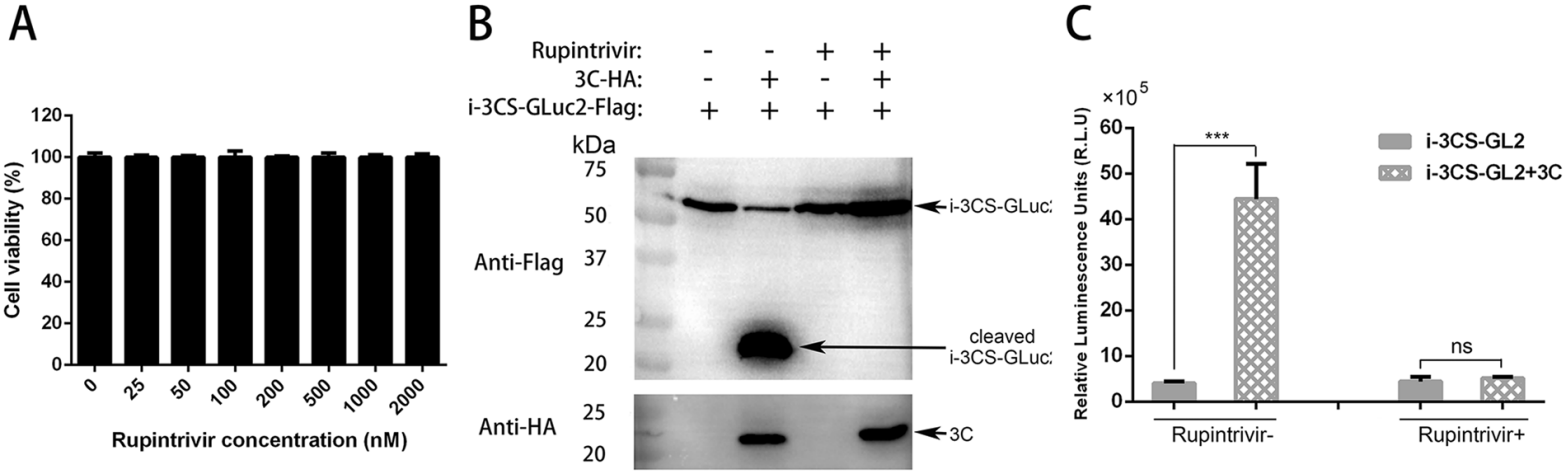
Monitoring the inhibition of EV71 3C^pro^ activity by Rupintrivir using the i-3CS-GLuc2 biosensor. (**A**) Cell viability of HEK293T cells treated with various concentrations of Rupintrivir determined by CCK-8 assay. The results are presented as the means ± SD of triplicate measurements. (**B**) Detection of the cleavage of i-3CS-GLuc2 by EV71 3C^pro^ treated with or without Rupintrivir via Western blot analysis. (**C**) Quantification of the EV71 3C^pro^ activity treated with or without Rupintrivir by luciferase assay. The results are presented as the means ± SD of triplicate measurements (Student’s t test; ns, nonsignificant, ***p < 0.001).

Rupintrivir (AG7088) is a compound originally developed as an irreversible inhibitor of human rhinovirus (HRV) 3C^pro^ ^26^. In 2013, scientists found that Rupintrivir also functions as a high-affinity inhibitor of EV71 3C^pro^ ^27^. Thus, we used Rupintrivir to determine whether our biosensor could be used to measure the inhibition of EV71 3C^pro^. To test whether Rupintrivir is toxic to human embryonic kidney 293 T (HEK293T) cells, the Cell Counting Kit-8 (CCK-8) assay was performed. Rupintrivir at concentrations from 25 nM to 2 μM showed no obvious toxicity to HEK293T cells (Fig. 3A). In the absence of Rupintrivir, EV71 3 C^pro^ cleaved and activated the biosensor i-3CS-GLuc2 to increase the GLuc signal from (4.07 ± 0.23) × 10^5^ to (44.40 ± 4.49) × 10^5^ (N = 3), with a SBR of 10.92 (Fig. 3C). However, upon treatment with 1 μM of Rupintrivir, EV71 3C^pro^ only led to a slight increase in signal from (4.42 ± 0.58) × 10^5^ to (5.16 ± 0.17) × 10^5^ (N = 3), with an SBR of 1.17. This result was highly consistent with Western blotting results (Fig. 3B), indicating that i-3CS-GLuc2 can accurately and sensitively monitor the inhibition event of EV71 3C^pro^ activity by Rupintrivir.

### Construction and characterization of an EV71 3C^pro^ activity reporter cell line

**Figure 4:**
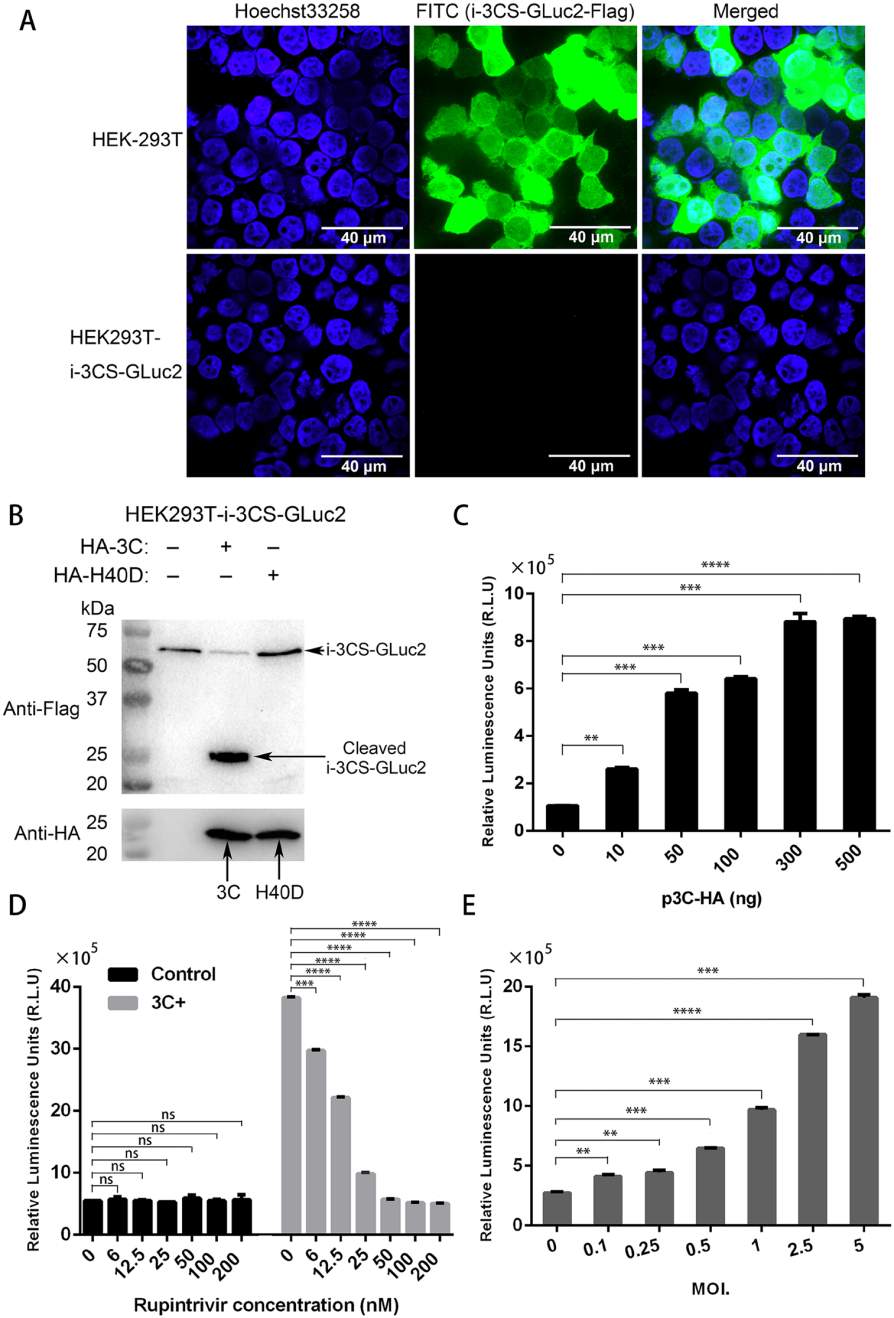
Construction and characterization of the EV71 3C^pro^ activity reporter cell line. (**A**) Immunofluorescence assay (IFA) of the control HEK293T cells and the EV71 3C^pro^ activity reporter cell line HEK293T-i-3CS-GLuc2 cells. The primary antibody is mouse anti-Flag antibody and the secondary antibody is FITC-labeled mouse anti-IgG antibody. Scale bar: 40 μm. (**B**) The cleavage of i-3CS-GLuc2 in HEK293T-i-3CS-GLuc2 cells by EV71 3C^pro^ or the catalytically inactive 3C^pro^ mutant H40D detected by Western blotting assays. The blots of Anti-HA were cropped and the full-length blots were displayed in Supplementary Information. (**C**) The dose effect of EV71 3C^pro^ on luciferase activity in HEK293T-i-3CS-GLuc2 cells. Cells were seeded in 12-well plates and transfected with various amounts of p3C-HA. The results are presented as the means ± SD of triplicate measurements (Student’s t test; **p < 0.01, ***p < 0.001, ****p < 0.0001). (**D**) The dose effect of Rupintrivir on luciferase activity in HEK293T-i-3CS-GLuc2 cells with and without 3C^pro^ expression. Cells were seeded in 12-well plates, transfected with and without 300 ng of p3C-HA and treated with Rupintrivir at various concentrations. The results are presented as the means ± SD of triplicate measurements (Student’s t test; ns, nonsignificant, ***p < 0.001, ****p < 0.0001). (**E**) The dose effect of the EV71 MOI value on luciferase activity in HEK293T-i-3CS-GLuc2 cells. The results are presented as the means ± SD of triplicate measurements (Student’s t test; **p < 0.01, ***p < 0.001, ****p < 0.0001).

To facilitate antiviral drug screening, a HEK293T cell line that stably expresses i-3CS-GLuc2-Flag was constructed as described in the Methods section. Immunofluorescence assays were performed to assess protein expression. As shown in Fig. 4A, HEK293T and HEK293T-i-3CS-GLuc2 cells were immunostained with fluorescein isothiocyanate (FITC) labeled Flag antibodies. Because no endogenous Flag-tagged proteins are expressed in HEK293T cells, no FITC fluorescence was observed. In contrast, HEK293T-i-3CS-GLuc2 cells showed strong FITC fluorescence, indicating that i-3CS-GLuc2 expressed stably and effectively in this cell line.

To confirm this result, the cell line was transfected with the empty vector pCAGGS, pHA-3C, and pHA-H40D, and cells were harvested and subjected to Western blotting assays. As shown in Fig. 4B, the biosensor i-3CS-GLuc2 was strongly expressed and could be cleaved by EV71 3C^pro^ in this cell line.

We next tested whether HEK293T-i-3CS-GLuc2 cells could be used to sensitively and accurately report EV71 3C^pro^ activity. We seeded HEK293T-i-3CS-GLuc2 cells in 12-well plates and transfected them with 0–500 ng of p3C-HA to test the dose relationship of EV71 3C^pro^ and Luc activity. As shown in Fig. 4C, a larger amount of EV71 3C^pro^ leads to stronger bioluminescence as predicted, indicating that HEK293T-i-3CS-GLuc2 cells can be used to report the activity of EV71 3C^pro^ in a quantitative manner.

We also tested the dose-dependent inhibition of EV71 3C^pro^ activity by Rupintrivir using our reporter cell line. As shown in Fig. 4D, the background bioluminescence generated by HEK293T-i-3CS-GLuc2 cells was not affected by the concentration of Rupintrivir. However, when EV71 3C^pro^ was expressed in the reporter cell line, the resulting bioluminescence increased to (38.28 ± 0.09) × 10^5^ and then decreased to (5.07 ± 0.04) × 10^5^ (N = 3) as the Rupintrivir concentration was increased from 0 nM to 200 nM.

To test whether EV71 infection will activate the biosensor within HEK293T-i3CS-GLuc2 cells, these cells were infected with an EV71 BrCr-TR strain at a multiplicity of infection (MOI) of 0–5. As shown in Fig. 4E, there was a good correlation between viral MOI and the bioluminescence produced by GLuc, indicating that the reporter cell line can be used to monitor EV71 infection.

### Optimization and validation of HEK293T-i-3CS-GLuc2 based antiviral drugs screening system

To develop a HEK293T-i-3CS-GLuc2 based antiviral drugs screening system, we first identified the appropriate MOI for viral infection. HEK293T-i-3CS-GLuc2 cells infected with the EV71 BrCr-TR strain at an MOI of 2.5 or 5 produced strong GLuc signals (Fig. 4E) and evident cytopathic effect (CPE) without detachment. Therefore, we selected an MOI of 4 in the following experiments.

We next used Rupintrivir to determine whether HEK293T-i-3CS-GLuc2 cells could be used as an anti-EV71 drug screening system. The reported half maximal inhibitory concentration (IC_50_) of Rupintrivir against the EV71 695 F strain (MOI = 0.1) is 14 nM in RD cells^27^. In this study, we examined the inhibitory effects of Rupintrivir at different concentrations against the EV71 BrCr-TR strain in our reporter cell line using Luc assays, the 50% tissue culture infectious dose (TCID_50_) assays, CPE observation, WB assays and quantitative reverse transcription PCR (RT-qPCR).

**Figure 5:**
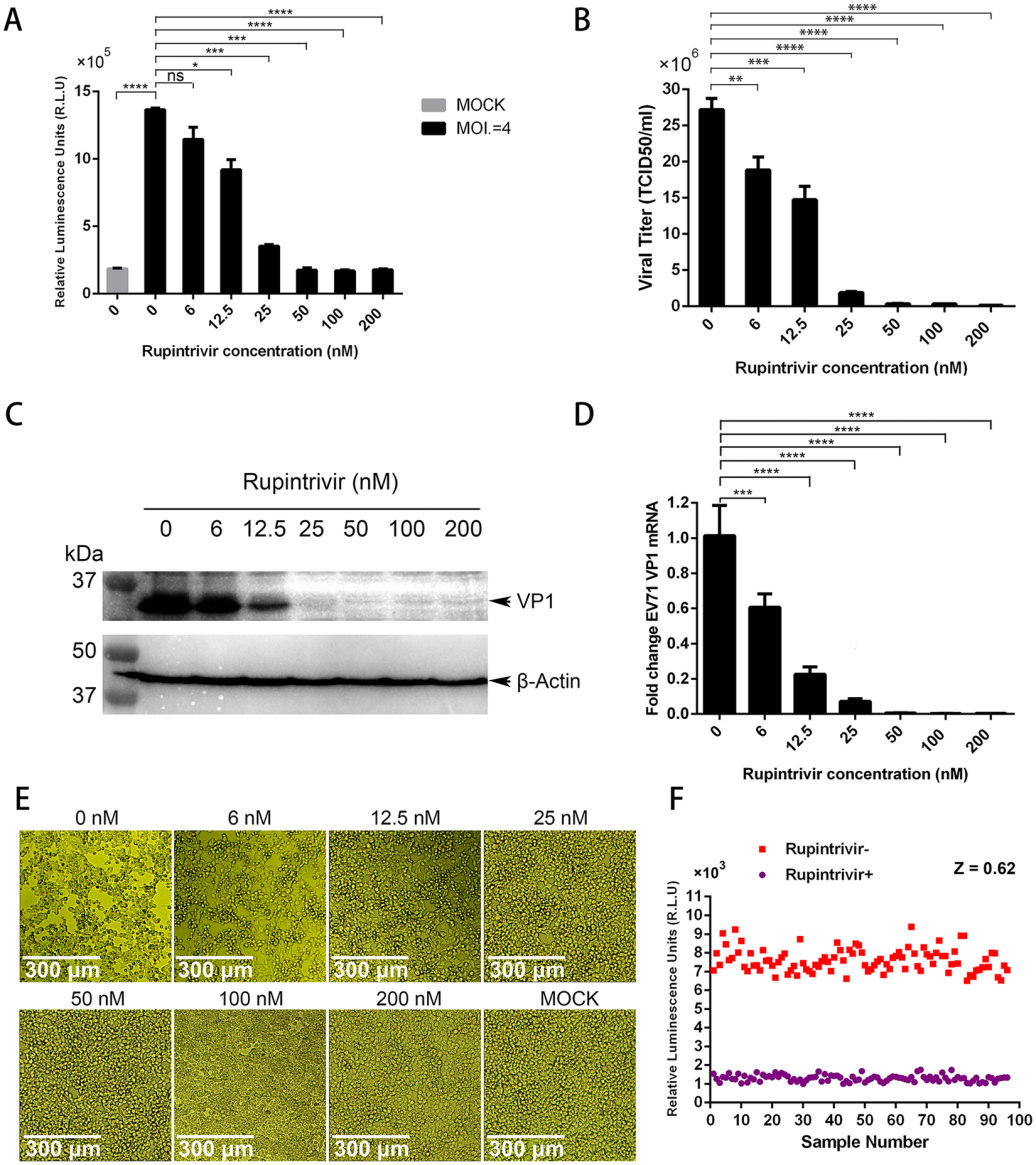
The anti-EV71 effect of Rupintrivir in HEK293T-i3CS-GLuc2 cells. In A-E, HEK293T-i-3CS-GLuc2 cells were infected with the EV71 BrCr-TR strain at an MOI = 4 and treated with Rupintrivir at 0, 6, 12.5, 25, 50, 100 and 200 nM, and tested at 24 hours post infection. (**A**) Dose effect of Rupintrivir on luciferase activity. The results are presented as the means ± SD of triplicate measurements (Student’s t test; ns, nonsignificant, *p < 0.05, ***p < 0.001, ****p < 0.0001). (**B**) Dose effect of Rupintrivir on viral titer detected by the TCID_50_ assay. The results are presented as the means ± SD of triplicate measurements (Student’s t test; **p < 0.01, ***p < 0.001, ****p < 0.0001). (**C**) The expression level of EV71 VP1 protein detected by Western blotting assays. The blots were cropped and the full-length blots were displayed in Supplementary Information. (**D**) Dose effect of Rupintrivir on the EV71 VP1 mRNA level detected by RT-qPCR. The results are presented as means ± SD of five measurements (Student’s t test; ***p < 0.001, ****p < 0.0001). (**E**) CPEs of EV71-infected cells treated with and without Rupintrivir at various concentration. Scale bar: 300 μm. (**F**) The data of a high-throughput assay using HEK293T-i3CS-GLuc2 cells accessing the inhibition effects of Rupintrivir. 1 × 10^5^ HEK293T-i-3CS-GLuc2 cells seeded in 96-well assay plates were infected with the EV71 BrCr-TR strain at an MOI of 4, and treated with 100 nM of Rupintrivir (Rupintrivir+) or methanol (Rupintrivir−). Bioluminescence was measured at 24 hours post infection.

The results of Luc assays were shown in Fig. 5A, the resulting bioluminescence of HEK293T-i-3CS-GLuc2 cells increased from (1.85 ± 0.04) × 10^5^ to (13.66 ± 0.09) × 10^5^ (N = 3) upon infection with the EV71 BrCr-TR strain and decreased to (1.78 ± 0.05) × 10^5^ (N = 3) as the Rupintrivir concentration increased from 0 nM to 200 nM, indicating the excellent anti-EV71 effect of Rupintrivir. Consistent with this result, the TCID_50_ value also decreased with an increasing Rupintrivir concentration in a dose-dependent manner (Fig. 5B). To further assess the anti-EV71 effects of Rupintrivir, we tested the expression of the viral structure protein VP1 with WBs. As shown in Fig. 5C, Rupintrivir treatment decreased the expression level of the VP1 protein in a dose-dependent manner. Furthermore, we tested the VP1 mRNA level using RT-qPCR. When treated with 200 nM Rupintrivir, the VP1 mRNA was only (0.31 ± 0.04)% (N = 5) of the level with no Rupintrivir treatment. As shown in Fig. 5E, the reporter cell line infected with the EV71 BrCr-TR strain presented evident CPEs 24 hours after infection. However, the cell morphology gradually returned to normal as the concentration of Rupintrivir was increased. The IC_50_ of Rupintrivir against the EV71 BrCr-TR strain on our reporter cell line was estimated to be around 12.5 nM based on these assays. The consistency of these experiments indicates that HEK293T-i-3CS-GLuc2 cells could be applied as an antiviral drug screening tool.

To validate whether HEK293T-i-3CS-GLuc2 cells could be used for high-throughput screening (HTS), the value of Z factor was determined. HEK293T-i-3CS-GLuc2 cells seeded in 96-well assay plates were infected with the EV71 BrCr-TR strain at an MOI of 4, then treated with Rupintrivir at 100 nM or with methanol as negative control. The assay data of representative samples were shown in Fig. 5F. According to the formula described in the previous research^28^, Z factor was calculated to be 0.62, indicating our system could provide excellent HTS assays (1 > Z > 0.5)^28^.

**Figure 6:**
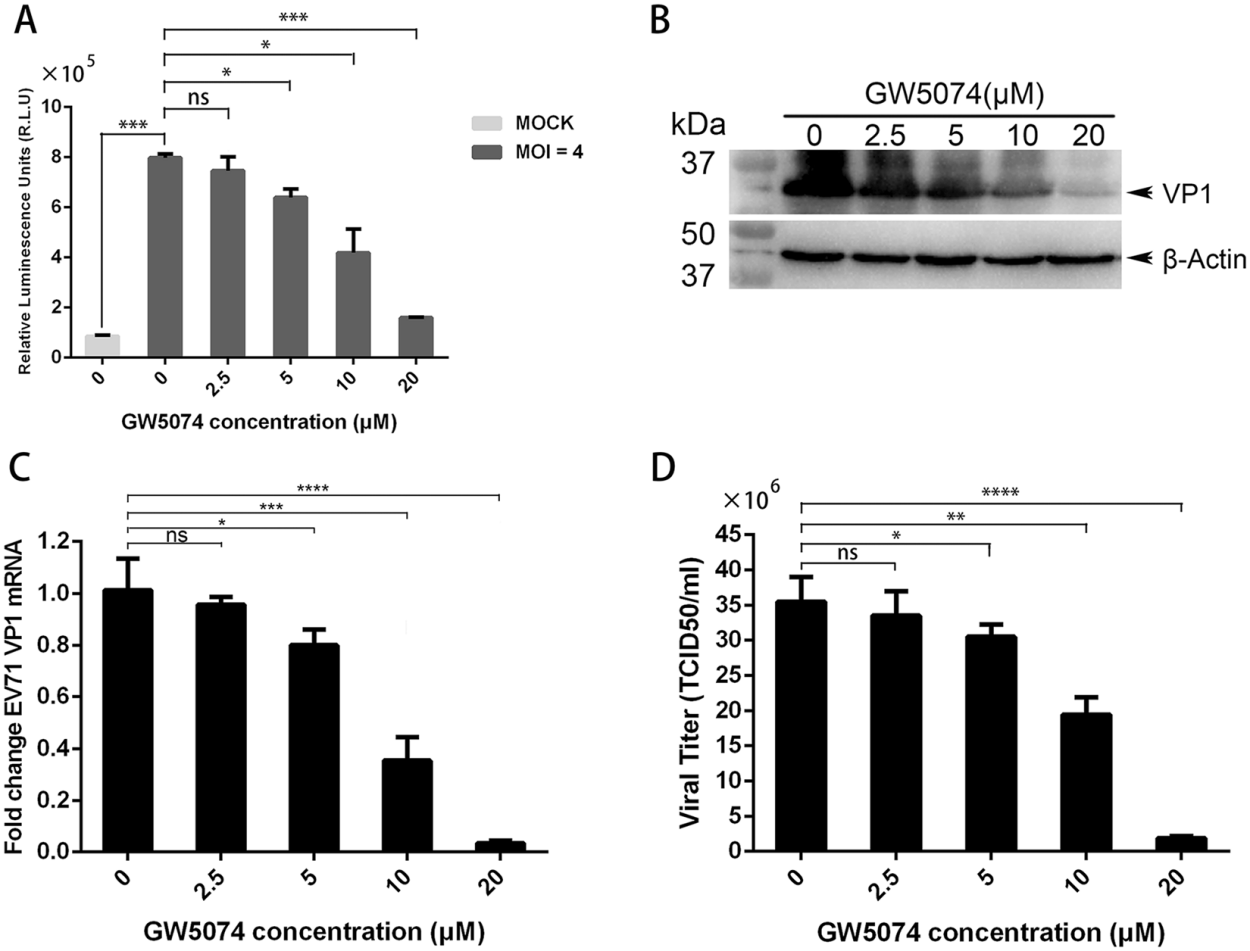
Anti-EV71 effect of GW5074 in HEK293T-i3CS-GLuc2 cells. In all experiments, HEK293T-i-3CS-GLuc2 cells were infected with the EV71 BrCr-TR strain at an MOI = 4 and treated with GW5074 at concentration of 0, 2.5, 5, 10, or 20 μM. Cells were tested at 24 hours post infection. (**A**) Dose effect of Rupintrivir on luciferase activity. The results are presented as the means ± SD of triplicate measurements (Student’s t test; ns, nonsignificant, *p < 0.05, ***p < 0.001). (**B**) The expression level of EV71 VP1 protein detected by Western blotting assays. (**C**) Dose effect of Rupintrivir on EV71 VP1 mRNA level detected by RT-qPCR. The results are presented as the means ± SD of five measurements (Student’s t test; ns, nonsignificant, *p < 0.05, ***p < 0.001, ****p < 0.0001) (**D**) Dose effect of Rupintrivir on viral titer detected by the TCID_50_ assays. The results are presented as the means ± SD of triplicate measurements (Student’s t test; ns, nonsignificant, *p < 0.05, **p < 0.01, ****p < 0.0001).

GW5074 [3-(3,5-Dibromo-4-hydroxybenzyliden)-5-iodo-1,3-dihydroindol-2-one] can suppress EV71 replication *in vitro* ^29^ and was used to validate an EV71 3C^pro^-dependent bioluminescence imaging assay for anti-viral screening in a previous study^30^. Herein, GW5074 was selected to further validate the efficiency of HEK293T-i-3CS-GLuc2 cells for screening anti-EV71 agents. First, the cytotoxicity of GW5074 on HEK293T cells was tested using CCK-8 assays. Treatment with GW5074 up to 20 μM showed no significant influence on cell viability (Fig. S3). To assess the anti-EV71 effects of GW5074, HEK293T-i-3CS-GLuc2 cells were infected with the EV71 BrCr-TR strain at an MOI of 4 and treated with different concentrations (0, 2.5, 5, 10 and 20 μM) of GW5074. As shown in Fig. S4, obvious CPEs were observed at 24 h post infection without GW5074 treatment, whereas the CPEs in the cells treated with GW5074 were inhibited in a dose-dependent manner. To assess the anti-EV71 effect of GW5074 using the i-3CS-GLuc2 biosensor, GLuc bioluminescence was measured 24 hours after infection. As shown in Fig. 6A, treatment with GW5074 significantly decreased the resulting bioluminescence in a dose-dependent manner. Additionally, WB assays showed that GW5074 treatment decreased EV71 VP1 expression (Fig. 5B) and RT-qPCR assay showed that GW5074 treatment decreased the EV71 VP1 mRNA level (Fig. 5C). The viral TCID_50_ was also decreased by GW5074 in a dose-dependent manner (Fig. 5D). The consistency of these experiments further indicates that HEK293T-i-3CS-GLuc2 cells could be used for antiviral drugs screening.

**Figure 7:**
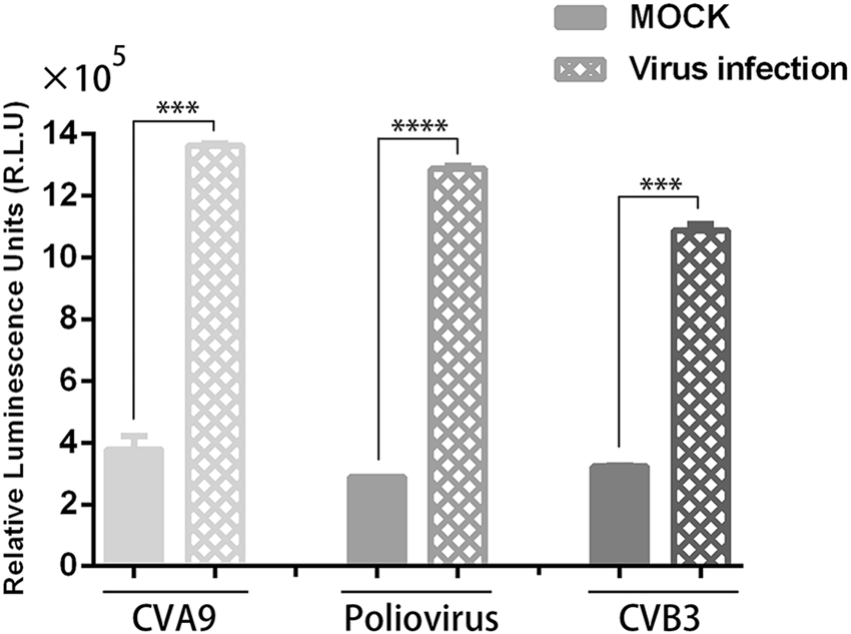
Monitoring the 3C^pro^ activity of CVA9, poliovirus, and CVB3 infection via HEK293T-i-3CS-GLuc2 cells. HEK293T-i-3CS-GLuc2 cells were infected with the CVA9 Griggs strain, the poliovirus vaccine strain Sabin 3, and the CVB3 Nancy strain at an MOI of 0.5 respectively. At 24 hours post infection, cells were harvested and subjected to luciferase assays. The results are presented as the means ± SD of triplicate measurements (Student’s t test; ***p < 0.001, ****p < 0.0001).

In the *Enterovirus* family, some viruses share the same 3C^pro^ cleavage site. Thus theoretically, the i-3CS-GLuc biosensor could be used for some other enteroviruses whose 3C^pro^ can recognize the EALFQ↓GPPK site. To test this possibility, the CVA9 Griggs strain, the CVB3 Nancy strain and the poliovirus vaccine strain Sabin 3 were used to infect HEK293T-i-3CS-GLuc2 cells at an MOI of 0.5. At 24 hours post infection, cells were harvested and subjected to luciferase assays. As shown in Fig. 7, all three viruses activated GLuc and increased bioluminescence. The SBRs of CVA9, CVB3, and poliovirus infection were 3.60, 3.36 and 4.45 (three replicates each), indicating that the biosensor is also effective for mearing 3 C^pro^ activity in other viruses of the *Enterovirus* family.

## Discussion

Considering the continual outbreaks and serious clinical manifestations of children and infants caused by EV71, the prevention and treatment of this virus are urgently desired. In this study, we developed and evaluated a Luc-based biosensor for viral protease activity. We demonstrated that EV71 3 C^pro^ can cleave and activate i-3CS-GLuc biosensor and thus report the 3C^pro^ activity in a quantitative manner.

Various viral protease activity reporter systems have been developed, most are based on *in vitro* purified enzymes and their substrates, and use different detection technologies such as the fluorescence resonance energy transfer (FRET)^31^. Briefly, peptides bearing protease cleavage sites and FRET donor-receptor pairs were used as substrates for *in vitro* purified proteases to analysis their activity. FRET signals were activated when cleavage of peptides separated the fluorescent donor from the acceptor. This strategy has been applied to multiple viral proteases for inhibitor screening, including NS2B-NS3 of West Nile Virus^32^ and 3C or 3C-like protease of Picornaviruses, Noroviruses, and Coronaviruses^33^. However, *in vitro* assays are often unable to reliably reflect the actual cleavage events that occur in complex milieus such as living cells and animals. In this study, both the biosensor and the reporter cell line allowed the monitoring of viral protease activity in living cells instead of *in vitro*. Numerous reports have demonstrated that the GLuc reporter can be used in small animal models as a marker to monitor different *in vivo* biological events such as tumor growth and proliferation, viral infection and replication as well as cell circulation^34–36^. Thus theoretically, our biosensor could also be used in animal models to monitor viral protease activity.

Compared with Western blotting and various enzymatic activity assays, Luc assays are highly sensitive and extremely easy to perform, requiring only minutes to accomplish. The ease of use and applicability as a transgene in cell-based assays of our biosensor render it a powerful tool to study viral protease cleavage events and screen antiviral drugs with high-throughput. In this study, we transduced the biosensor i-3CS-GLuc2 into HEK293T cells to generate a reporter cell line and verified that this cell line could be applied as a high-throughput antiviral screening tool. This biosensor could also be transduced into other cell types to generate additional reporter cell lines and monitor viral protease activity in various cells including all types of nerve cells.

Moreover, in addition to enteroviruses including EV71, CVA9, CVB3, and poliovirus, by changing the cleavage site, this reporter could also be used to detect the protease activity of other viruses. However, some restrictions should be taken into consideration when it was applied to other viral proteases. The sensor protein should be expressed in the same intracellular location with the viral protease to ensure it could be cleaved to generated active GLuc. In our case, both proteins were expressed in the cytosol. But some viral proteases may localize to different subcellular structures, for example, dengue virus NS2B-NS3 is bound to the ER membrane. The lack of physical interaction would make the protease activity detection impossible.

In summary, we have established a Luc-based biosensor for EV71 3C^pro^ activity and developed a high-throughput antiviral agent screening system on the basis of this biosensor. This system is highly sensitive, easily operated and accurately quantifiable, providing a powerful tool for monitoring viral protease activity and for performing the high-throughput screening of antiviral drugs in living cells. In theory, this biosensor could be expanded to other proteases of many other type of viruses, and be applied in animal models. Therefore, this biosensor should be extremely useful for studying viral proteases and screening novel antiviral agents.

## Methods

### Cells and viruses

HEK293T cells and African green monkey kidney epithelial cells (Vero, CCL-81, American Type Culture Collection) were cultured in Dulbeco’s modified Eagle’s medium (DMEM, Thermo Fisher Scientific). Human rhabdomyosarcoma cells (RD, CCL-136, American Type Culture Collection) were grown in minimal essential medium (MEM, Thermo Fisher Scientific). All cells were maintained in medium supplemented with 10% fetal bovine serum (FBS, Gibco) at 37 °C with 5% CO_2_.

The EV71 BrCr-TR strain was obtained from the Institute of Medical Biology, Chinese Academy of Medical Science. The CVA9 Griggs strain was obtained from the Institute of Biomedical Engineering, Chinese Academy of Medical Sciences, and Peking Union Medical College. The CVB3 Nancy strain was obtained from Wuhan University. All viruses were amplified in Vero cells. Virus titer was measured by the TCID_50_ in RD cells using the Reed–Muench formula^37^.

### Antibodies and reagents

Mouse anti-Flag, mouse anti-HA, mouse anti-β-Actin antibodies and GW5074 were purchased from Sigma-Aldrich (St. Louis, MO). EV71 VP1 polyclonal antibody was prepared by immunizing rabbits with his-tagged VP1 protein. Coelenterazine was obtained from Promega. RIPA lysis buffer and the protease inhibitor phenylmethylsulfonyl fluoride (PMSF) were purchased from Beyotime (Jiangsu, China). Rupintrivir was purchased from Santa Cruz Biotechnology. Lipofectamine® 2000 was purchased from Thermo Fisher Scientific.

### Plasmid construction

The mouse IL–1β-3CS DNA fragment was synthesized by Tianyihuiyuan Company (Wuhan, China). The GLuc encoding plasmid pGLuc-Basic was purchased from NEB. The pi-GLuc-Flag, pi-3CS-GLuc1-Flag and pi-3CS-GLuc2-Flag constructs were generated by overlap PCR from mouse IL–1β-3CS DNA fragment and pGLuc-Basic and inserted into the pCAGGS vector. The Super PiggyBac transposase expression vector and PiggyBac (PB) Dual Promoter vector were purchased from System Biosciences. The pPB-i-3CS-GLuc2-Flag was generated via overlap PCR and In-Fusion Cloning. DNA sequences of iGLuc, i-3CS-GLuc1, and i-3CS-GLuc2 were given in the supplementary information.

### Cleavage assay detected by Western blotting assays

HEK293T cells were seeded in 6-well plates and transfected with corresponding plasmids using Lipofectamine® 2000 Reagent. At 24 hours post transfection, cells were lysed with RIPA lysis buffer (50 mM Tris, pH 7.4, 150 mM NaCl, 1% NP-40, 0.5% sodium deoxycholate, 0.1% SDS) plus protease inhibitor PMSF and subjected to SDS-PAGE. Proteins were separated and transferred to polyvinylidene difluoride (PVDF) membranes. The membranes were then blocked and incubated with respective primary antibodies and horseradish peroxidase (HRP)-conjugated secondary antibodies (Pierce). After washing, membranes were incubated with the Immobilon Western chemiluminescent HRP substrate (Millipore) and subjected to the Bio-Rad Imaging System for imaging analysis.

### Cleavage assay detected by luciferase assay

HEK293T cells were seeded in 24-well plates and transfected with corresponding plasmids using Lipofectamine® 2000 Reagent. At 24 hours post transfection, cells were lysed with 100 μL of passive lysis buffer (Promega) per well. 25 μL of lysate was mixed with 25 μL of coelenterazine (5 μM). The resulting bioluminescence was measured on a GloMax®20/20 Luminometer (Promega).

### Assay of EV71 3C^pro^ inhibition by Rupintrivir using plasmid expression

For Western blotting assays, HEK293T cells were seeded in 6-well plates and transfected with corresponding plasmids. At 7 hours post transfection, cells were treated with 1 μM of Rupintrivir or methanol. At 30 hours post transfection, cells were harvested and subjected to Western blotting as described above.

For luciferase assay, HEK293T cells were seeded in 24-well plates and transfected with corresponding plasmids. At 7 hours post transfection, cells were treated with 1 μM of Rupintrivir or methanol. At 30 hours post transfection, cells were harvested and subjected to luciferase assay as described above.

### Construction of i-3CS-GLuc2 stable expression cell line

HEK293T cells were seeded in 6-well plates and transfected with pPB-i-3CS-GLuc2-Flag and a Super PB transposase expression vector. At 30 hours post transfection, selection was performed by adding 4 μg/ml of puromycin in the culture medium. Colonies were further passaged in puromycin-containing medium to get stable cell line. After detection of i-3CS-GLuc2 expression with indirect immunofluorescence and Western Blotting, cell lines were cultured in medium containing 4 μg/ml of puromycin all the time to maintain their stability.

### Immunofluorescence assay

HEK293T and HEK293T-i-3CS-GLuc2 cells were seeded in confocal dishes (NEST). At 24 hours post seeding, cells were washed, fixed with 4% formaldehyde, permeabilized with 2% Triton-X100, blocked with PBS buffer containing 2% BSA and 5% normal goat serum (Beyotime), and immunostained with anti-Flag monoclonal antibodies and FITC-labeled secondary antibodies. After washing, cell nuclei were counterstained with Hoechst 33258 (Beyotime) and visualized on a PerkinElmer UltraView VOX system using a Nikon Ti microscope.

### Cleavage assay in HEK293T-i-3CS-GLuc2 cells using Western blotting assays

HEK293T-i-3CS-GLuc2 cells were seeded in 6-well plates and transfected with corresponding plasmids. At 24 hours post transfection, cells were harvested and subjected to Western blot as described above.

### Dose effect of 3C^pro^ on the HEK293T-i-3CS-GLuc2 cells detected by luciferase assays

HEK293T-i3CS-GLuc2 were seeded in 12-well plates and transfected with 0–500 ng of p3C-HA using Lipofectamine® 2000. At 24 hours post transfection, cells were harvested and subjected to Luc assays as described above.

### Assay of EV71 3C^pro^ inhibition by Rupintrivir in HEK293T-i3CS-GLuc2 cells

HEK293T-i-3CS-GLuc2 cells were seeded in 12-well plates and transfected with empty vector or 300 ng of p3C-HA. At 6 hours post-transfection, cells were treated with Rupintrivir at different concentrations. At 24 hours post transfection, cells were lysed and subjected to Luc assays as described above.

### Viral infection experiments on HEK293T-i-3CS-GLuc2 cells

HEK293T-i3CS-GLuc cells were seeded in 24-well plates and infected with the EV71 BrCr-TR strain at an MOI of 0–5, or they were infected with the CVA9 Griggs strain, the CVB3 Nancy strain, or the poliovirus vaccine strain Sabin 3 (Leon 12a1b, GenBank: X00596.1) at an MOI of 0.5. At 24 hour post infection, cells were harvested and subjected to luciferase assays as described above.

### Assay of EV71 inhibition by Rupintrivir and GW5074 in HEK293T-i-3CS-GLuc2 cells

HEK293T-i3CS-GLuc2 cells were seeded in 24-well plates, infected with the EV71 BrCr-TR strain at an MOI of 4 and incubated with various concentrations (0–200 nM) of Rupintrivir or various concentrations (0–20 μM) of GW5074 1 hour after infection. At 24 hour post infection, cells were visualized under a microscope (OLYMPUS) to record any CPEs. Also, the GLuc activity of each well was measured as described above and the VP1 expression level of each well was detected by WB assays as described above. Additionally, the cells were disrupted by three sequential freeze-thaw cycles between −80 °C and 37 °C. The cellular debris was removed by centrifugation. Viral titers were determined by the TCID_50_ assay in RD cells using the Reed-Muench formula. For RT-qPCR assays, total RNA was extracted from cells by using TRIzol reagent (Ambion). First cDNA strands were generated using PrimeScript^TM^ RT reagent Kit with gDNA Eraser (TaKaRa). Realtime PCR was conducted with SYBR Green Master Mix (Bio-Rad) on a CFX Connect Real-Time PCR Detection System from Bio-Rad.

### Z factor determination

1 × 10^5^ HEK293T-i-3CS-GLuc2 cells were seeded in 96-well assay plates (Corning), infected with the EV71 BrCr-TR strain at an MOI of 4, and treated with 100 nM of Rupintrivir or with methanol as negative control. At 24 hours post infection, cells were lysed with 40 μL of passive lysis buffer per well and disrupted by one freeze-thaw between −80 °C and 37 °C. Then 40 μL of coelenterazine (5 μM) were added to the lysates and bioluminescence measurements were made using a Multimode Microplate Reader (Varioskan Flash, Thermo Fisher, Finland). The value of Z factor was calculated using the formula described in a previous report^28^.

## Electronic supplementary material

Supplementary information
