# Supplementary material for: Development of a luciferase-based biosensor to assess enterovirus 71 3C protease activity in living cells
Source: Sci Rep. 2017 Sep 4;7:10385. doi: 10.1038/s41598-017-10840-x (PMC5583365; doi:10.1038/s41598-017-10840-x)

Development of a luciferase-based biosensor to assess enterovirus 71 3C protease activity in living cells

Yuan Zhang[1], Xianliang Ke[2], Caishang Zheng[2], Yan Liu[1], Li Xie[1], Zhenhua Zheng[1] and Hanzhong Wang[1] *

[1]Key Laboratory of Special Pathogens and Biosafety, Center for Emerging Infectious Diseases, Wuhan Institute of Virology, Chinese Academy of Sciences, Wuhan 430071, China

[2] Guangzhou Institute of Pediatrics, Guangzhou Women and Children Medical Center, Guangzhou, 510623, china

*E-mail: wanghz@wh.iov.cn. Phone: +86-027-8719-7295.

Supplementary Information

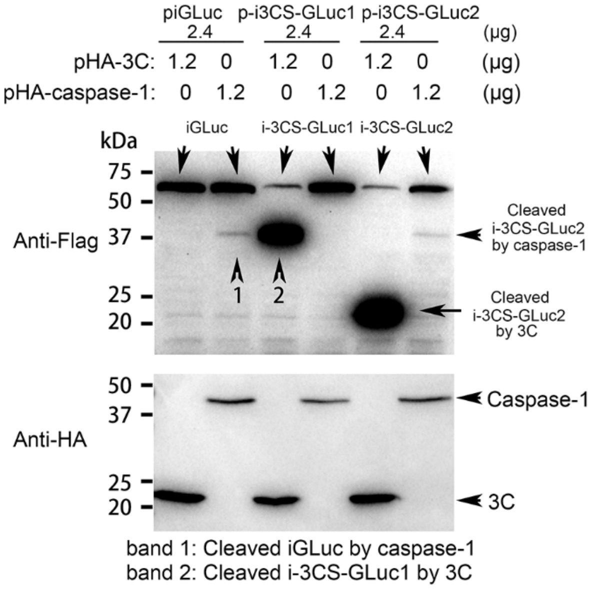

Figure S1. Cleavage of iGLuc, i-3CS-GLuc1, and i-3CS-GLuc2 by EV71 $3C^{pro}$ and mouse caspase-1 detected by Western blotting assays. HEK293T cells were seeded in 6-well plates and transfected with 2.4 μg of piGLuc-Flag, pi-3CS-GLuc1-Flag, or pi-3CS-GLuc2-Flag and 1.2 μg of pHA-3C or pHA-caspase-1. Cells were harvested and subjected to WBs at 24 hours post transfection.

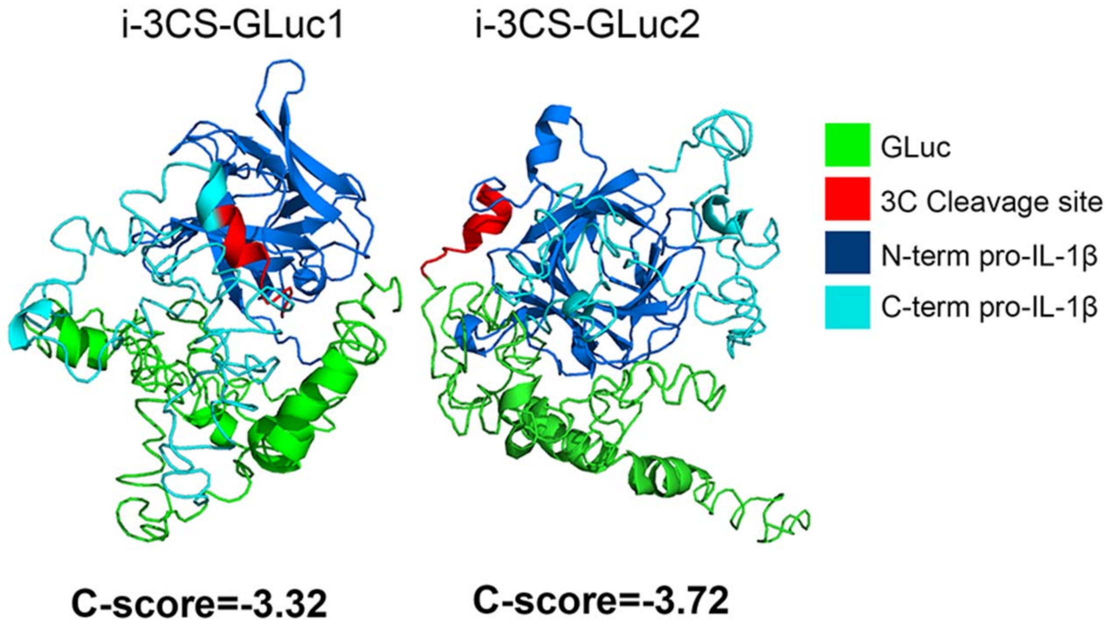

Figure S2. Top ranked model with the highest cluster size of i-3CS-GLuc1 (left) and i-3CS-GLuc2 (right) predicted by I-TASSER online server. C-score for each model is shown in the figure. C-score indicates the confidence of predicted models, typically in the range of [-5,2], a higher C-core implicates higher confidence of the I-TASSER prediction.

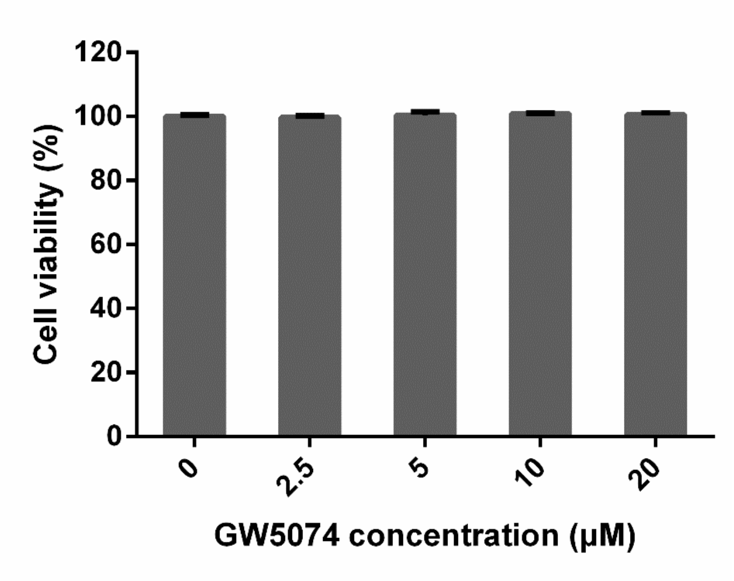

Figure S3. Cell viability of HEK293T treated with GW5074 at various concentrations. HEK293T cells were treated with GW5074 at various concentrations, the cell viability was measured by CCK-8 using corresponding untreated cells as controls (set as 100%). The results are presented as the means ± SD of triplicate measurements.

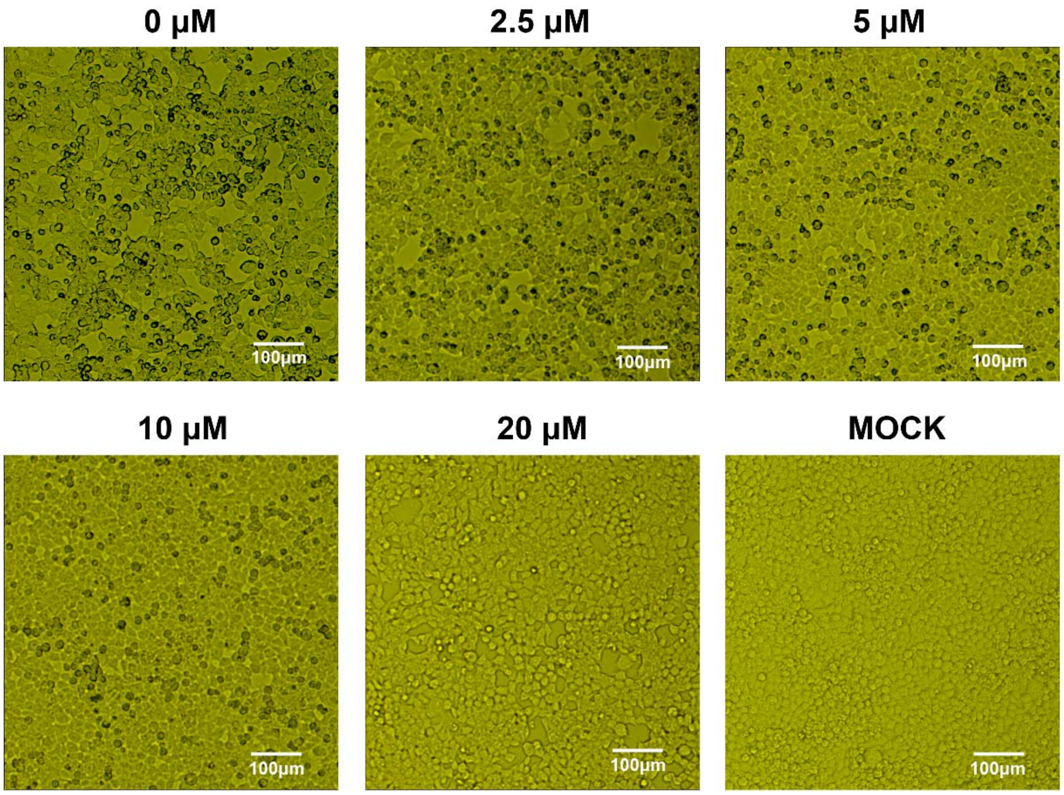

Figure S4. CPEs in HEK293T-i3CS-GLuc2 cells infected with EV71 and treated with GW5074 at virous concentrations. HEK293T-i3CS-GLuc2 cells were infected with the EV71 BrCr-TR strain at an MOI of 4 and treated with GW5074 at various concentrations. At 24 hours post infection, cells were visualized under a microscope. One representative experiment out of three is shown. Scale bar: 100 μm.

Table S1. Construct sequences of iGLuc, i-3CS-GLuc1, and i-3CS-GLuc2

NAME	SEQUENCE	COMMENT	BACKBONES
iGLuc-Flag	atggcaactgttcctgaactcaactgtgaaatgccaccttttgacagtga<br>tgagaatgacctgttctttgaagttgacggaccccaaaagatgaaggg<br>ctgcttccaaacctttgacctgggctgtcctgatgagagcatccagcttc<br>aaatctcgcagcagcacatcaacaagagcttcaggcaggcagtatca<br>ctcattgtggctgtggagaagctgtggcagctacctgtgtctttcccgtgg<br>accttccaggatgaggacatgagcaccttcttttccttcatctttgaagaa<br>gagcccatcctctgtgactcatgggatgatgatgataacctgTTAGT<br>CTGCGATGTAcccattagacaactgcactacaggctccgagat<br>gaacaacaaaaaagcctcgtgctgtcggacccatatgagctgaaag<br>ctctccacctcaatggacagaatatcaaccaacaagtgatattctccat<br>gagctttgtacaaggagaaccaagcaacgacaaaatacctgtggcct<br>tgggcctcaaaggaaagaatctatacctgtcctgtgtaatgaaagacg<br>gcacacccaccctgcagctggagagtgtggatcccaagcaataccc<br>aaagaagaagatggaaaaacggtttgtcttcaacaagatagaagtca<br>agagcaaagtggagtttgagtctgcagagttccccaactggtacatca<br>gcacctcacaagcagagcacaagcctgtcttcctgggaaacaacagt<br>ggtcaggacataattgacttcaccatggaatccgtgtcttccGGATC<br>CACTAGTGGCGAGGCCAAGCCCACCGAGAACA<br>ACGAAGACTTCAACATCGTGGCCGTGGCCAGCA<br>ACTTCGCGACCACGGATCTCGATGCTGACCGCG<br>GGAAGTTGCCCGGCAAGAAGCTGCCGCTGGAG<br>GTGCTCAAAGAGATGGAAGCCAATGCCCGGAAA<br>GCTGGCTGCACCAGGGGCTGTCTGATCTGCCTG<br>TCCCACATCAAGTGCACGCCCAAGATGAAGAAG<br>TTCATCCCAGGACGCTGCCACACCTACGAAGGC<br>GACAAAGAGTCCGCACAGGGCGGCATAGGCGA<br>GGCGATCGTCGACATTCCTGAGATTCCTGGGTT<br>CAAGGACTTGGAGCCCATGGAGCAGTTCATCGC<br>ACAGGTCGATCTGTGTGTGGACTGCACAACTGG<br>CTGCCTCAAAGGGCTTGCCAACGTGCAGTGTTC<br>TGACCTGCTCAAGAAGTGGCTGCCGCAACGCTG<br>TGCGACCTTTGCCAGCAAGATCCAGGGCCAGGT<br>GGACAAGATCAAGGGGGCCGGTGGTGACgattac<br>aaggatgacgacgataag	Blue = pro–IL-1β<br>Yellow = caspase-1 cleavage site<br>Black = linker<br>Green = GLuc<br>Brown = Flag tag	pCAGGS
i-3CS-GLuc1-Flag	Atggcaactgttcctgaactcaactgtgaaatgccaccttttgacagtga<br>tgagaatgacctgttctttgaagttgacggaccccaaaagatgaaggg<br>ctgcttccaaacctttgacctgggctgtcctgatgagagcatccagcttc<br>aaatctcgcagcagcacatcaacaagagcttcaggcaggcagtatca<br>ctcattgtggctgtggagaagctgtggcagctacctgtgtctttcccgtgg<br>accttccaggatgaggacatgagcaccttcttttccttcatctttgaagaa<br>gagcccatcctctgtgactcatgggatgatgatgataacctggaagca	Blue = pro–IL-1β<br>Red = 3C^{pro} cleavage site<br>Black = linker	pCAGGS

	ctctttcaaggaccccctaaacccattagacaactgcactacaggctcc<br>gagatgaacaacaaaaaagcctcgtgctgtcggacccatatgagctg<br>aaagctctccacctcaatggacagaatatcaaccaacaagtgatattct<br>ccatgagctttgtacaaggagaaccaagcaacgacaaaatacctgtg<br>gccttgggcctcaaaggaaagaatctatacctgtcctgtgtaatgaaag<br>acggcacacccaccctgcagctggagagtgtggatcccaagcaata<br>cccaaagaagaagatggaaaaacggtttgtcttcaacaagatagaa<br>gtcaagagcaaagtggagtttgagtctgcagagttccccaactggtac<br>atcagcacctcacaagcagagcacaagcctgtcttcctgggaaacaa<br>cagtggtcaggacataattgacttcaccatggaatccgtgtcttccGG<br>ATCCACTAGTGGCGAGGCCAAGCCCACCGAGAA<br>CAACGAAGACTTCAACATCGTGGCCGTGGCCAG<br>CAACTTCGCGACCACGGATCTCGATGCTGACCG<br>CGGGAAGTTGCCCGGCAAGAAGCTGCCGCTGG<br>AGGTGCTCAAAGAGATGGAAGCCAATGCCCGGA<br>AAGCTGGCTGCACCAGGGGCTGTCTGATCTGCC<br>TGTCCCACATCAAGTGCACGCCCAAGATGAAGA<br>AGTTCATCCCAGGACGCTGCCACACCTACGAAG<br>GCGACAAAGAGTCCGCACAGGGCGGCATAGGC<br>GAGGCGATCGTCGACATTCCTGAGATTCCTGGG<br>TTCAAGGACTTGGAGCCCATGGAGCAGTTCATC<br>GCACAGGTCGATCTGTGTGTGGACTGCACAACT<br>GGCTGCCTCAAAGGGCTTGCCAACGTGCAGTGT<br>TCTGACCTGCTCAAGAAGTGGCTGCCGCAACGC<br>TGTGCGACCTTTGCCAGCAAGATCCAGGGCCAG<br>GTGGACAAGATCAAGGGGGCCGGTGGTGACgatt<br>acaaggatgacgacgataag	Green = GLuc<br>Brown = Flag tag	
i-3CS-GLuc2-Flag	atggcaactgttcctgaactcaactgtgaaatgccaccttttgacagtga<br>tgagaatgacctgttctttgaagttgacggaccccaaaagatgaaggg<br>ctgcttccaaacctttgacctgggctgtcctgatgagagcatccagcttc<br>aaatctcgcagcagcacatcaacaagagcttcaggcaggcagtatca<br>ctcattgtggctgtggagaagctgtggcagctacctgtgtctttcccgtgg<br>accttccaggatgaggacatgagcaccttcttttccttcatctttgaagaa<br>gagcccatcctctgtgactcatgggatgatgatgataacctgTTAGT<br>CTGCGATGTAcccattagacaactgcactacaggctccgagat<br>gaacaacaaaaaagcctcgtgctgtcggacccatatgagctgaaag<br>ctctccacctcaatggacagaatatcaaccaacaagtgatattctccat<br>gagctttgtacaaggagaaccaagcaacgacaaaatacctgtggcct<br>tgggcctcaaaggaaagaatctatacctgtcctgtgtaatgaaagacg<br>gcacacccaccctgcagctggagagtgtggatcccaagcaataccc<br>aaagaagaagatggaaaaacggtttgtcttcaacaagatagaagtca<br>agagcaaagtggagtttgagtctgcagagttccccaactggtacatca<br>gcacctcacaagcagagcacaagcctgtcttcctgggaaacaacagt<br>ggtcaggacataattgacttcaccatggaatccgtgtcttccgaagcact	Blue = pro–IL-1β<br>Yellow = caspase-1<br>Red = $3C^{pro}$ cleavage site<br>Green = GLuc<br>Brown = Flag tag	pCAGGS

	ctttcaaggaccccctaaaGAGGCCAAGCCCACCGAGAACAACGAAGACTTCAACATCGTGGCCGTGGCCAGCAACTTCGCGACCACGGATCTCGATGCTGACCGCGGGAAGTTGCCCGGCAAGAAGCTGCCGCTGGAGGTGCTCAAAGAGATGGAAGCCAATGCCCGGAAAGCTGGCTGCACCAGGGGCTGTCTGATCTGCCTGTCCCACATCAAGTGCACGCCCAAGATGAAGAAGTTCATCCCAGGACGCTGCCACACCTACGAAGGCGACAAAGAGTCCGCACAGGGCGGCATAGGCGAGGCGATCGTCGACATTCCTGAGATTCCTGGGTTCAAGGACTTGGAGCCCATGGAGCAGTTCATCGCACAGGTCGATCTGTGTGTGGACTGCACAACTGGCTGCCTCAAAGGGCTTGCCAACGTGCAGTGTTCTGACCTGCTCAAGAAGTGGCTGCCGCAACGCTGTGCGACCTTTGCCAGCAAGATCCAGGGCCAGGTGGACAAGATCAAGGGGGCCGGTGGTGACgattacaaggatgacgacgataag		

Full-length blots for Figure 2D, Anti-HA

The red mark indicates the cropped part reported in Figure 2D, Anti-HA.

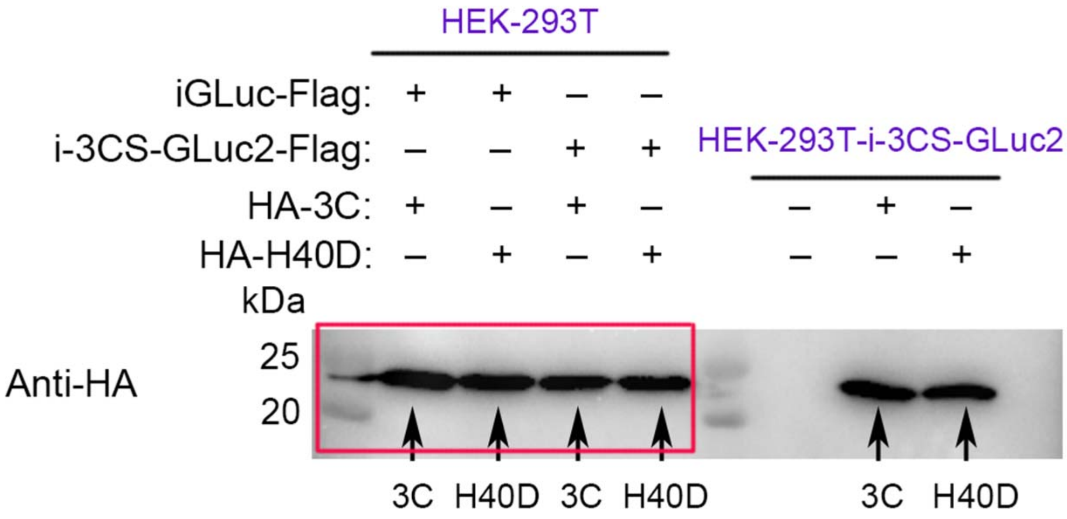

Full-length blots for Figure 4B, Anti-HA

The red mark indicates the cropped part reported in Figure 4B, Anti-HA.

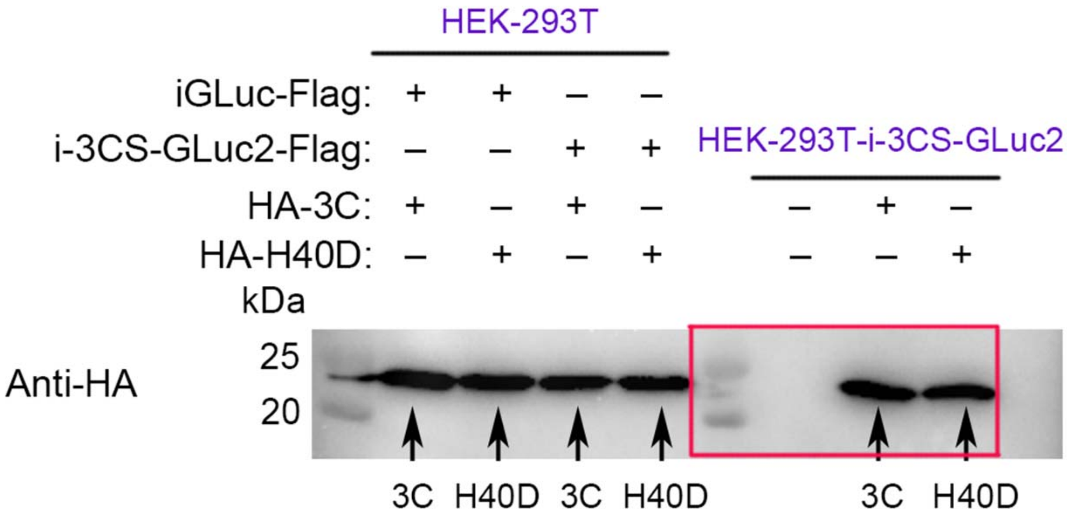

Full-length blots for Figure 5C

The red mark indicates the cropped part reported in Figure 5C.

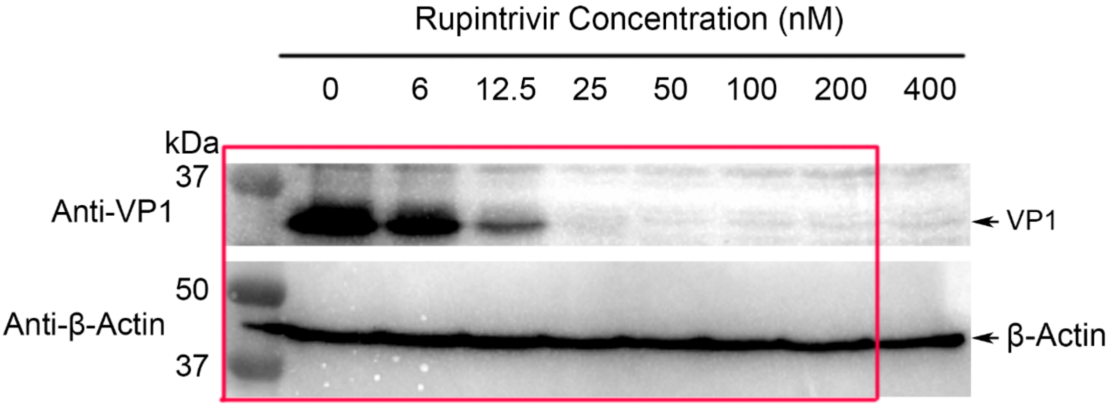

Supplement: Supplementary file 1 — Supplementary information [file 41598_2017_10840_MOESM1_ESM.pdf]
